# Prediction of Judkins Left Catheter Size during Left Transradial Coronary Angiography by Simple Chest Radiographic and Echocardiographic Index

**DOI:** 10.3390/medicina57101124

**Published:** 2021-10-18

**Authors:** Seong-Soon Kwon, Byoung-Won Park, Duk-Won Bang, Min-Ho Lee, Min-Su Hyon, Seong-Soo Lee

**Affiliations:** Department of Internal Medicine, Division of Cardiology, Soonchunhyang University Seoul Hospital, Seoul 04401, Korea; kwon.seongsoon@schmc.ac.kr (S.-S.K.); schbdw@schmc.ac.kr (D.-W.B.); neoich@schmc.ac.kr (M.-H.L.); mshyon@schmc.ac.kr (M.-S.H.); seongsoo.lee@schmc.ac.kr (S.-S.L.)

**Keywords:** catheter, transradial coronary angiography, mediastinum

## Abstract

*Background and Objectives:* Appropriate catheter selection when conducting transradial coronary angiography (CAG) helps shorten examination time, preventing vascular complications and lowering medical expense. However, catheter selection is made based on the practitioner’s experience in almost all cases. Therefore, we undertook this study to define radiologic and echocardiographic indices that would enable physicians to anticipate appropriate catheter selection. *Materials and Methods:* This is a retrospective study of 244 undergoing transradial diagnostic CAG at an established center from February 2006 to April 2014. Patients who successfully underwent angiography with a JL3.5 catheter were defined as the control group, and patients who successfully underwent angiography after the catheter was replaced with a JL4.0 or higher were defined as the switched group. To identify predictors for appropriate catheter selection, radiologic and echocardiographic indices were analyzed. *Results:* A total of 122 patients in the switched group and 122 patients in the control group were analyzed in this study. Average age was 64.65 ± 8.6 years. In the radiographic index, the switched group exhibited a significantly higher mediastinal-thoracic ratio (0.27 ± 0.05 vs. 0.23 ± 0.03, *p* < 0.001. Additionally, the mediastinal-cardiac ratio was significantly greater in the switched group (0.50 ± 0.08 vs. 0.45 ± 0.05, *p* < 0.001). Aortic root diameter, which is used here as the echocardiographic index, was significantly larger in the switched group compared to the control group (34.94 ± 4.18 mm vs. 32.66 ± 3.99 mm, *p* < 0.001). In the multivariable logistic regression model, mediastinal-cardiac ratio (OR 5.197, 95% CI 2.608–10.355, *p* < 0.001) and increased aortic root (OR 2.115, 95% CI 1.144–3.912, *p* = 0.017) were significantly associated with catheter change. *Conclusions:* Mediastinal-cardiac ratio and aortic root diameter provide helpful and effective indices for appropriate catheter selection during transradial coronary angiography.

## 1. Introduction

Appropriate catheter selection during transradial coronary angiography helps shorten examination time, preventing vascular complications and decreasing medical expense.

**Figure 1:**
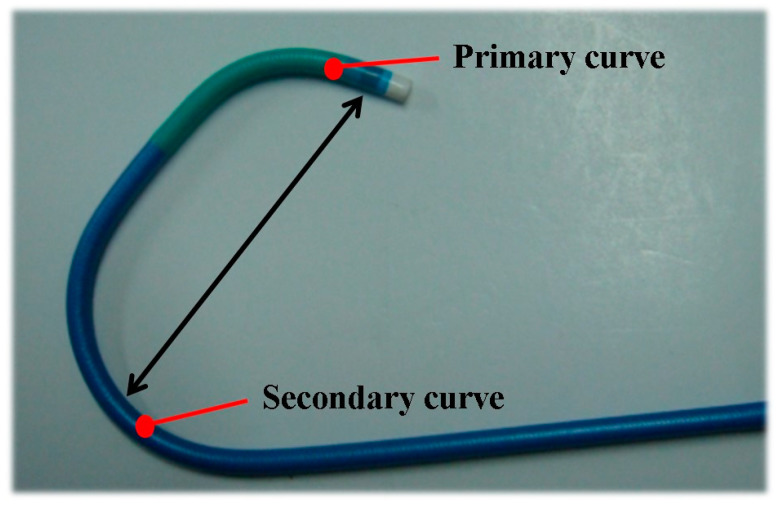
Tip of the Judkins left catheter. The numbers (e.g., JL 3.5) indicating the size of the Judkins left catheter means the length of the primary to the second curve.

A Judikins Left (JL) catheter is often used in transradial coronary angiography. The JL catheter is composed of both a primary and secondary curve (Figure 1). During coronary angiography, the distal primary curve enables the catheter to engage smoothly in the left coronary opening, whereas the proximal secondary curve was designed to maintain catheter stability by reaching the opposite side of the aorta. When the aorta is expanded, stability can be maintained only if a catheter with a longer curve is selected [1].

Since there is no well-defined method of catheter selection suitable for an expanded aorta, selection depends on the practitioner’s prior experiences in almost all cases. Therefore, we evaluated radiologic and echocardiographic indices which reflect aortic expansion in order to anticipate appropriate catheter selection.

## 2. Methods

### 2.1. Study Population

Patient data and angiograms were retrospectively collected from inpatients undergoing transradial diagnostic CAG at a tertiary referral public hospital from February 2006 to April 2014. Our institution was a high-volume transradial center (>150 percutaneous coronary intervention by radial artery per year). Four operators undertook radial coronary angiography. All operators were experienced interventional cardiologist (more than 4 years of experience each). We included patients who underwent successful angiography made possible by changing to a JL 4.0 or larger catheter after angiography failure with a JL 3.5 catheter. The control group was made up of patients who achieved successful transradial coronary angiography using a JL 3.5 catheter. Patients in the two groups were matched on a 1:1 basis according to gender and age.

To predict appropriate catheter selection, radiologic and echocardiographic indices were analyzed prospectively. For the radiologic index, cardio-thoracic ratio, mediastinal-thoracic ratio, and mediastinal-cardiac ratio were measured using simple chest radiography. Aortic root diameter was measured for the echocardiographic index. 

### 2.2. Data Collection

As factors pertinent to catheter selection, height, weight, presence of major risk factors for cardiovascular disease (hypertension, diabetes, dyslipidemia), radiologic index measured with simple chest radiography, and echocardiographic index were analyzed prospectively. 

**Figure 2:**
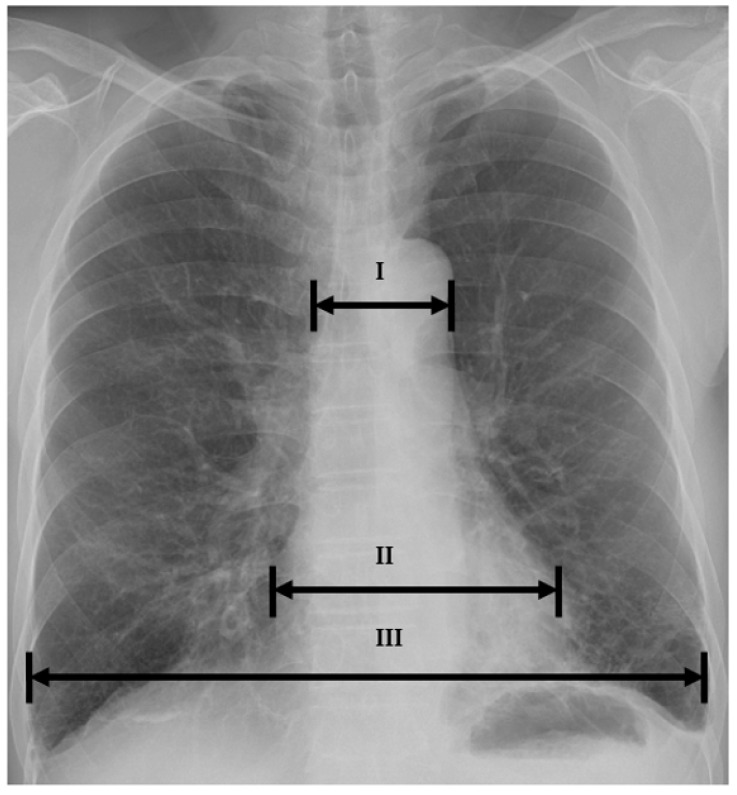
Calculation of radiologic parameters on simple chest radiography. Cardio-thoracic ratio = II/III; mediastinal-thoracic ratio = I/III; mediastinal-cardiac ratio = I/II; I = mediastinum width; II = maximal transverse diameter of heart; III = maximal transverse diameter of chest.

**Figure 3:**
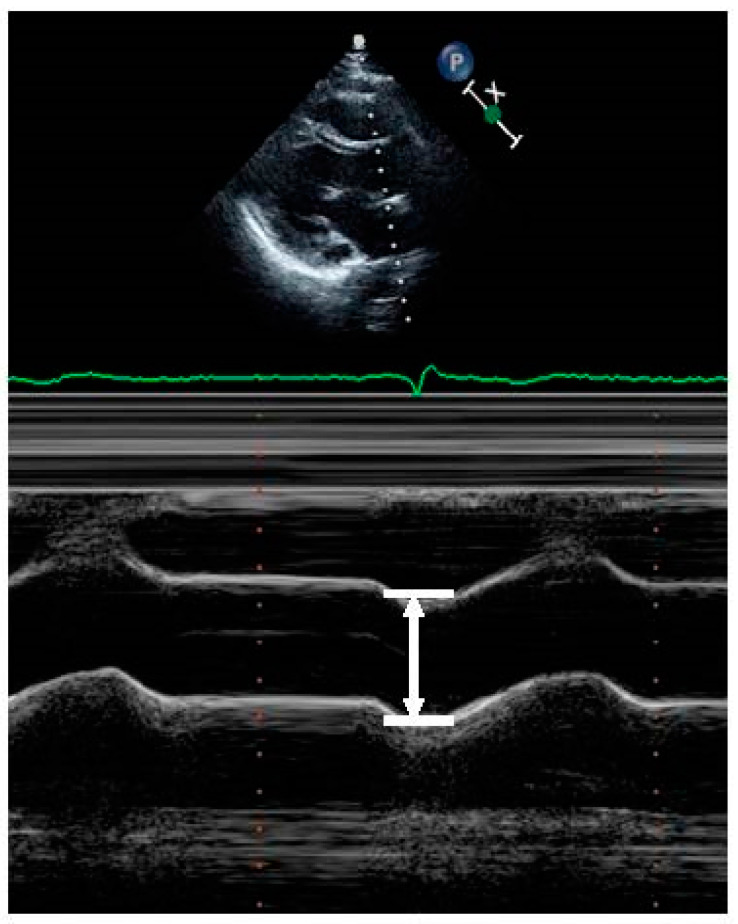
Measurement of aortic root diameter on M-mode echocardiograph. For measurement of aortic root diameter, place the cursor over the aortic valve cusps. Identify diastole when start of the QRS on the EKG, immediately before the aortic valve opens and measure the aortic root diameter.

For the radiologic index, cardio-thoracic ratio, mediastinal-thoracic ratio, and mediastinal-cardiac ratio were confirmed from a simple chest radiography (posteroanterior projection) conducted before or within 6 months of the procedure. Mediastinum width was measured by calculating the distance from the right mediastinum to the left mediastinum at the aortic cushion level of the upper mediastinum. Cardio-thoracic ratio was measured with a unidimensional calculation (maximum diameter of heart/maximum diameter of chest) [2]. Mediastinal-thoracic ratio was measured by the diameter of mediastinum dividing the by the maximum diameter of chest. Mediastinal-cardiac ratio was measured by the diameter of mediastinum dividing the chest by the maximum diameter of heart [3] (Figure 2). Aortic root diameter was measured using M mode in parasternal long axis view on echocardiography (Figure 3) [4].

This study was performed in accordance with the ethical guidelines of the 1975 Declaration of Helsinki and was approved by the Institutional Review Board of our institution (SCHUH 2020-02-007, Approved date: 14 February 2020). Due to the retrospective nature of this study, informed consent was not required.

### 2.3. Statistical Analysis

Continuous variables are presented as mean ± standard deviation and compared between study groups using the independent *t*-test. Categorical variables are expressed as frequencies and percentages and compared using the Pearson’s Chi-squared test. In addition, once a predictive factor was proven to be statistically significant, sensitivity and specificity were analyzed using a receiver operating characteristic (ROC) curve and then the cut-off value was selected. An odds ratio (OR) was calculated from each cut-off value. A *p*-value <0.05 was considered statistically significant. All statistical analyses were performed using SPSS version 21.0 (IBM Co., Armonk, NY, USA).

### 2.4. Data Availability

The data associated with the paper are not publicly available but are available from the corresponding author on reasonable requests.

## 3. Results

A total of 122 patients in the switched group and 122 patients in the control group were analyzed in this study. The average age of all groups was 64.65 ± 8.6 years and 203 (83.20%) were male. There was no significant difference in height and weight between the two groups. The switched group showed a higher frequency of hypertension (82.0% vs. 70.5%, *p* = 0.050) (Table 1). 

In the radiographic index, there was no significant difference in cardio-thoracic ratio calculated using simple chest radiography (0.51 ± 0.08 vs. 0.51 ± 0.06, *p* = 0.064). However, the switched group showed a significantly higher mediastinal-thoracic ratio (0.27 ± 0.05 vs. 0.23 ± 0.03, *p* < 0.001). Additionally, a significant increase was observed in the mediastinal-cardiac ratio in the switched group (0.50 ± 0.08 vs. 0.45 ± 0.05, *p* < 0.001). Aortic root diameter, which is used as the echocardiographic index, was significantly larger in the switched group compared to the control group (34.94 ± 4.18 mm vs. 32.66 ± 3.99 mm, *p* < 0.001) (Table 2).

**Figure 4:**
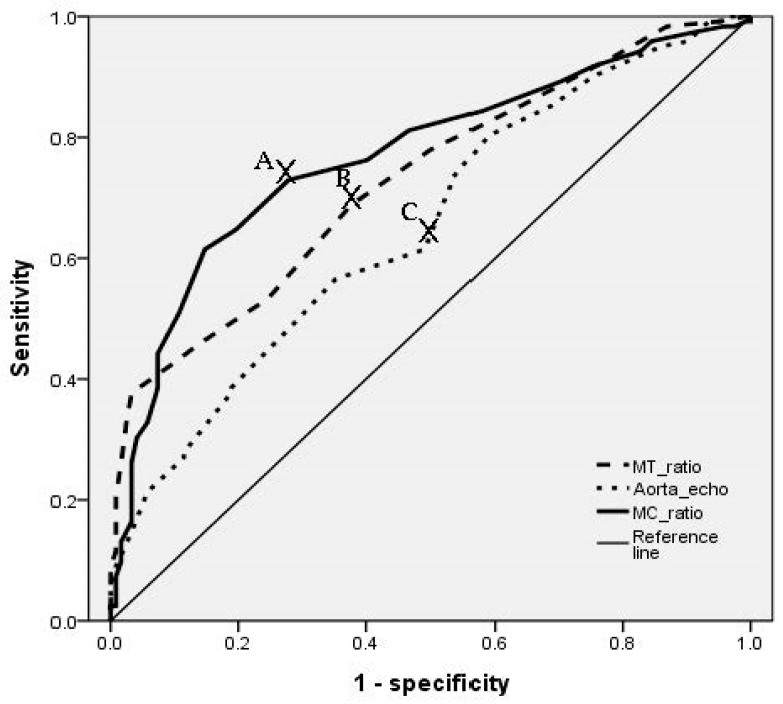
Receiver operating characteristics (ROC) curve of radiologic and echocardiographic parameters for prediction of catheter selection. (**A**) Cut off value of mediastinal-cardiac ratio on chest X-ray–0.47. (SN: 0.689, SP: 0.562, OR: 5.61, AUC: 0.769, 95% CI: 0.708~0.829). (**B**) Cut off value of mediastinal-thoracic ratio on chest X-ray–0.23. (SN: 0.734, SP: 0.566, OR: 1.91, AUC: 0.730, 95% CI: 0.667~0.792). (**C**) Cut off value of aortic root diameter on echocardiography ratio–34 mm. (SN: 0.591, SP: 0.578, OR: 2.24, AUC: 0.652, 95% CI: 0.583~0.720). SN: sensitivity, SP: specificity, OR: odds ratio, AUC: area under curve, CI: confidence interval.

Where there were statistically meaningful differences in the simple chest radiographic and echocardiographic results, cut-off values were defined using an ROC curve (Figure 4). When the mediastinal-thorax ratio was 0.23, sensitivity was 0.734, specificity was 0.566, OR was 1.91 (95% confidence interval (CI): 1.040–3.480), and area under the curve (AUC) was 0.730 (95% CI: 0.667–0.792). When the mediastinal-cardiac ratio was 0.47, sensitivity was 0.689, specificity was 0.562, OR was 5.61 (95% CI: 2.853–11.104), and AUC was 0.769 (95% CI: 0.708–0.829). When aortic root diameter was 34 mm, sensitivity was 0.591, specificity was 0.578, OR was 2.24 (95% CI: 1.290–4.112), and AUC was 0.652 (95% CI: 0.583–0.720) (Table 3). In the multivariable logistic regression model wherein all these predictors were considered, mediastinal-cardiac ratio (OR 5.197, 95% CI 2.608–10.355, *p* < 0.001) and increased aortic root (OR 2.115, 95% CI 1.144–3.912, *p* = 0.017) were significantly associated with catheter change (Table 4).

## 4. Discussion 

Transradial coronary angiography was first conducted by Campeau in 1989 [5], and transradial coronary intervention was first used by Kiemeneij and Laarman in 1993 [6]. Today, transradial coronary angiography and intervention are frequently used because they have a lower risk of hemorrhage and mortality compared to a transfemoral approach [7]. There is a learning curve for these interventions and new practitioners may be insufficiently trained [8]. Guidelines for appropriate catheter selection after successful puncture during transradial coronary angiography would have several benefits, such as shortened examination time, prevention of vascular complications, and decreased financial burden [9]. As previously described, the JL catheter is designed to allow for an easier approach to the left coronary artery. JL catheters are available in several sizes depending on the length of the primary and secondary curves, 3 cm to 6 cm (Figure 1). As aortic diameter increases, a catheter with a longer curve is recommended [1]. 

During left coronary artery angiography via the femoral artery without an expanded aortic root, a JL 4.0 catheter is preferred. However, for transradial coronary angiography, a JL 3.5 catheter is more useful for anatomical reasons. It was reported that of 234 patients who underwent transradial coronary angiography, 184 (79%) underwent left coronary angiography with a JL 3.5 catheter. Of these, 170 procedures were successfully completed [10]. However, there was no specific explanation as to why the catheter was changed for the other 14 patients. Several studies have reported that aortic expansion including aortic dissection can be predicted by measuring the width of the mediastinum using simple chest radiographic imaging [11,12]. According to the *American Journal of Roentgenology*, the ratio between the mediastinum and the chest can be used as an index for aortic expansion: a ratio greater than 0.25 indicates an expanded aorta with a sensitivity of 95% and a specificity of 75% [3].

In this study, we suggest that radiological imaging of the mediastinal-thoracic ratio, mediastinal-cardiac ratio, and aortic root showing a wide mediastinum indicate that catheters longer than JL 3.5 are more suitable. A cut-off value for each index was calculated as follows: mediastinal-thoracic ratio, 0.23; mediastinal-cardiac ratio, 0.47; and aortic root diameter, 34 mm. By the multivariable logistic regression model, mediastinal-cardiac ratio (OR 5.197) and increased aortic root (OR 2.115) were good parameters for selecting a catheter.

This study has some limitations. First, this is a retrospective analysis and, although the control group and the switched group were selected on a 1:1 basis, the absolute number of individuals in the target group was relatively low. Second, outside of the mentioned predictive indices, several variables, such as technical problems caused by changing of catheters, might influence the final result. Third, some measurement error is to be expected; for example, imaging might not be appropriately conducted when the patient performs inspiration during simple chest radiography, which is a radiographic index, or the boundary of the mediastinum could be vague. Additionally, the aorta can be twisted, especially in older patients, and simple chest radiography may not reflect this. Lastly, it is highly likely that applying these factors will not be necessary for already experienced operators, and the results of this study are considered to be applicable only to beginners in the radial approach.

This study revealed that simple indices measured by chest radiography and echocardiography are useful as predictors for appropriate catheter selection. It is necessary to conduct a well-designed prospective study using the predictive indices which can accurately measure the sizes of the mediastinum, thorax, and heart. 

## 5. Conclusions

Mediastinal-cardiac ratio and aortic root diameter provide helpful and effective indices for appropriate catheter size selection during transradial coronary angiography.

## Figures and Tables

**Table 1:** Baseline characteristics of the patients.

	Switched Group (*n* = 122)	Control Group (*n* = 122)	*p* Value
Age	64.84 ± 9.4	64.45 ± 7.9	0.726
Male	102 (83.6%)	101(82.8%)	>0.99
Height (cm)	161.9 ± 9.4	160.5 ± 13.1	0.182
Weight (kg)	64.6 ± 14.8	65.1 ± 11.5	0.769
Hypertension	100 (82.0%)	86 (70.5%)	0.050
Diabetes mellitus	56 (57.7%)	41 (42.3%)	0.067
Dyslipidemia	56 (52.3%)	51 (47.7%)	0.606

**Table 2:** Comparison of simple chest radiographic and echocardiographic parameters between groups.

Parameters		Switched Group (*n* = 122)	Control Group (*n* = 122)	*p* Value
I	Cardio-thoracic ratio	0.51 ± 0.08	0.51 ± 0.06	0.064
	Mediastinal-thoracic ratio	0.27 ± 0.05	0.23 ± 0.03	<0.001
	Mediastinal-cardiac ratio	0.50 ± 0.08	0.45 ± 0.05	<0.001
II	Aortic root diameter (mm)	34.94 ± 4.18	32.66 ± 3.99	<0.001

I: chest x-ray; II: echocardiography.

**Table 3:** Cut off values of simple chest radiographic and echocardiographic parameters for prediction of catheter selection.

	Value	SN	SP	AUC (95% CI)	OR (95% CI)
Mediastinal-thoracic ratio	0.23	0.689	0.562	0.730 (0.667–0.792)	1.91 (1.040–3.480)
Mediastinal-cardiac ratio	0.47	0.734	0.566	0.769(0.708–0.829)	5.61(2.853–11.104)
Aortic root (mm)	34	0.591	0.578	0.652 (0.583–0.720)	2.24 (1.290–4.112)

SN: sensitivity, SP: specificity, OR: odds ratio, AUC: area under curve, CI: confidence interval.

**Table 4:** Multivariable logistic regression analysis to predict catheter selection.

	Adjusted Odds Ratio (95% Confidence Interval)	*p* Value
Mediastinal-cardiac ratio	5.197 (2.608–10.355)	<0.001
Mediastinal-thoracic ratio	1.839 (0.967–3.497)	0.063
Aortic root (mm)	2.115 (1.144–3.912)	0.017

The AUC (95% CI) value of the model including these three variables was 0.708 (0.647–0.770).

## Data Availability

The data underlying this article cannot be shared publicly to protect the privacy of individuals that participated in the study. The data will be shared on reasonable request to the corresponding author.
